# Decoding the formation of diverse petal colors of *Lagerstroemia indica* by integrating the data from transcriptome and metabolome

**DOI:** 10.3389/fpls.2022.970023

**Published:** 2022-09-07

**Authors:** Sidan Hong, Jie Wang, Qun Wang, Guozhe Zhang, Yu Zhao, Qingqing Ma, Zhiqiang Wu, Jin Ma, Cuihua Gu

**Affiliations:** ^1^College of Landscape and Architecture, Zhejiang A&F University, Hangzhou, China; ^2^Zhejiang Provincial Key Laboratory of Germplasm Innovation and Utilization for Garden Plants, Zhejiang A&F University, Hangzhou, China; ^3^Key Laboratory of National Forestry and Grassland Administration on Germplasm Innovation and Utilization for Southern Garden Plants, Zhejiang A&F University, Hangzhou, China; ^4^Guangdong Laboratory for Lingnan Modern Agriculture, Genome Analysis Laboratory of the Ministry of Agriculture, Agricultural Genomics Institute at Shenzhen, Chinese Academy of Agricultural Sciences, Shenzhen, China; ^5^Kunpeng Institute of Modern Agriculture, Foshan, China; ^6^College of Science, Health, Engineering and Education, Food Futures Institute, Murdoch University, Murdoch, WA, Australia

**Keywords:** anthocyanin, breeding, color diversity, flower color, *Lagerstroemia indica*, metabolome

## Abstract

*Lagerstroemia indica* has great economic value due to its ecological, medicinal, and ornamental properties. Because its bloom color is one of the most essential characteristics, research into its color development is a hot topic. In this study, five representative colored cultivars were chosen, each representing a different color, such as white, red, pink, violet, and purple. Fully bloomed flowers were used to detect flavonoids in the petals. Anthocyanin is the main factor for the color formation of *L. indica*. 14 anthocyanins were discovered among the 299 flavonoids. Among 14 anthocyanins, malvidin-3,5-di-*O*-glucoside varied greatly among four colored samples and is the main contributor to color diversity. Transcriptome sequencing revealed that compared to white flowers, Anthocyanin pathway genes appear to be more active in colored samples. Analyzing the correlation network between metabolites and differential expressed genes, 53 key structural genes, and 24 TFs were detected that may play an essential role in the formation of color in *L. indica* flowers. Among these, the differential expression of *F3′5′H* and *F3′H* between all samples are contributors to color diversity. These findings lay the foundation for discovering the molecular mechanism of *L. indica* flower color diversity.

## Introduction

Flavonoids/anthocyanins, carotenoids, and betalains are the most common pigments that provide plants their colors (Tanaka et al., 2008). Carotenoids are lipid-soluble chemicals that contribute to yellow to red color and are extensively distributed in seed plants that engage in photosynthesis (Szabolcs, 1989). Betalains are water-soluble chemicals that give an orange to red-purple coloration and are exclusively found in the order Caryophyllales (Timoneda et al., 2019). In addition, anthocyanins that belong to Flavonoids are the most common essential pigment and play a key role in flower color formation (Winkel Shirley, 2001). There are six major forms of anthocyanidins in the plant world based on the substituents of the benzene ring: delphinidin, petunidin, cyanidin, malvidin, pelargonidin, and peonidin (Zhao and Tao, 2015; Yonekura Sakakibara et al., 2019). Different types and content of anthocyanins in plant tissues result in the unique color of the flowers ranging from orange/red to purple/blue. Besides, anthocyanins are vital for heredity in plants because of attracting pollinators and animals that can help with broadcasting pollen and seeds (Landi et al., 2015). Moreover, it can also defend plants from biotic or abiotic stresses (Dao et al., 2011; Liang and He, 2018). Therefore, plants with abundant anthocyanins are more likely to be favored by consumers.

Pathway genes and transcription factors (TF) that regulate anthocyanin biosynthesis are relatively conserved in many plants (Hichri et al., 2011). One molecule of *p*-coumaroly-CoA and three molecules of malonyl-CoA are converted to naringenin chalcone by catalysis of chalcone synthase (CHS) (Cain et al., 1997). Then, chalcone isomerase (CHI) rapidly converts naringenin chalcone to naringenin. Subsequently, naringenin is converted to eriodictyol or pentahydroxy flavone by the catalysis of F3′H or F3*′*5*′*H respectively (Koes et al., 2005). Following catalysis of a series of downstream enzymes, dihydroflavonol reductase (DFR), anthocyanin synthase (ANS), *O*-methyltransferases (OMT), and UDP glycosyltransferase (UGT) various types of anthocyanins are produced (Irmisch et al., 2019). Thus, the flow direction of the carbon flux in the pathway branch is very important, especially in the downstream branch from naringenin. F3H has a direct link to the production of pelargonidin, which can result in red flowers (Lukačin et al., 2000). In cornflower, the varied expression of gene *F3′H* and *DFR* decides the different concentrations of cyanidin and pelargonidin respectively, and thus influences the formation of flower color (Deng et al., 2019). According to some studies, delphinidin is a major pigment of most blue flowers, F3*′*5*′*H is the key enzyme of delphinidin synthesis. Therefore, F3*′*5*′*H may have strong relevance in forming the blue-hued flowers (Tanaka, 2006). When compared to wild peas with violet-colored flowers, the absence of expression of *F3′5′H* in pink flowers leads to a reduction in the delphinidin content (Moreau et al., 2012). There is a similar issue in *Lyceum* and chrysanthemums (He et al., 2013). MYB-bHLH-WD40 (MBW) transcription complexes are critical for controlling the flavonoid pathway. The control mechanism of these three genes is critical for efficiently regulating the expression of key structural genes. R2R3-myeloblastosis (MYB), basic helix-loop-helix (bHLH), and WD40 protein make up the MBW. bHLH is a protein that is generally conserved. *AtTT8* can regulate the expression of DFR and ANS in the capsule of seeding in *Arabidopsis* which contributes to the increased accumulation of anthocyanins (Nesi et al., 2000). Moreover, WD40 is related to the synthesis of floral pigments (Brueggemann et al., 2010; Payyavula et al., 2013). Of all three types of TFs, MYB is the most significant. MYBs, as essential transcription factors, bind directly to others to stimulate gene expression. Four MYBs (*AtPAP1*, *AtPAP2*, *AtMYB113*, and *AtMYB114*) are identified in *Arabidopsis* that influence the accumulation of anthocyanins (Borevitz et al., 2000; Teng et al., 2005; Stracke et al., 2007; Gonzalez et al., 2008). *VvMYBA1* and *VvMYBA2* in grape (Walker et al., 2007), *MdMYB10*, and *MdMYB110a* in apple (Espley et al., 2007; Chagné et al., 2012), and *PpMYB10.1* are reported to be responsible for anthocyanin synthesis (Rahim et al., 2014). In addition, 49 candidate MYBs were identified in leaves of *L. indica* and it is shown that they had a favorable influence on the regulation of the flavonoid-anthocyanin pathway (Qiao et al., 2019).

*Lagerstroemia indica* is a species of the *Lagerstroemia* genus that originated in China and is now widespread in most Chinese cities (Liu et al., 2008). It is a type of garden tree with high decorative value and many wonderful ornamental characteristics, such as a long flowering period, distinctive floral shapes, and lovely features (Pounders et al., 2007). Among others, the color of its petals with a wide hue (white, pink, red, purple, violet, and their combined colors) is most prominent. However, consumers increasingly want flowers with more unique colors such as real blue, and current cultivars are no longer able to meet market demand (Cabrera, 2004). Some research has been conducted on pigment composition and a preliminary molecular investigation. Four types of anthocyanins are detected in the flowers (delphinidin 3-*O*-glucoside, petunidin 3-*O*-glucoside, malvidin 3-*O*-glucoside, and cyanidin 3-*O*-glucoside) (Zhang et al., 2008) and seven R2R3-MYB transcription factors (TFs) were identified as probable regulators of flower color (Yu et al., 2021). However, neither the metabolic nor molecular foundation for the *L. indica* color diversity is entirely clear, and the control of the anthocyanin pathway requires further investigation.

In summary, the key anthocyanins and the molecular mechanism causing the color diversity of crape myrtle are unclear. In this study, we performed metabolomics and transcriptomics analyses of five typical colored petal tissue of crape myrtle cultivars at full bloom stages. Differentially expressed metabolites (DEMs) and differentially expressed genes (DEGs) were identified and analyzed. In addition, the molecular mechanisms of color diversity of *L. indica* petals would be discussed.

## Materials and methods

### Plant materials

**FIGURE 1:**
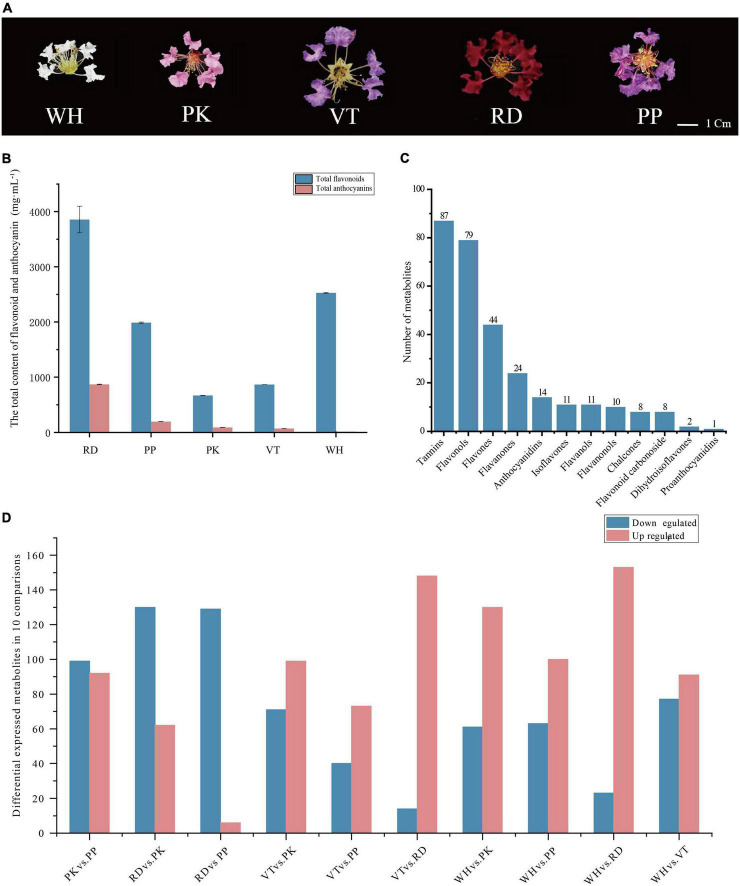
Phenotypes of comparisons, composition, and total content of flavonoids and anthocyanins among five *Lagerstroemia indica* petals. **(A)** Phenotypes of *L. indica* petals. **(B)** The total content of flavonoid and anthocyanin in five samples. **(C)** Classification and number of all flavonoids detected. **(D)** Number of differential accumulated metabolites between all comparisons.

In this investigation, five different *L. indica* cultivars were employed. The petal colors of the five cultivars were “Natchez” (white, WH), “Lihongtianyuan” (pink, PK), and “Honghuaguifei” (red, RD), “Zihuaguifei” (purple, PP), and “Lanzi” (violet, VT). All plant materials used were acquired from the Germplasm Resource Nursery of Crape Myrtle in the School of Landscape and Architecture, Zhejiang Agriculture and Forestry University (located at long. 118°51′ to 119°52′ E, lat. 29°56′ to 30°23′ N). Petal samples of five cultivars from the full bloom stage were collected (Figure 1A) from 8 am to 10 am. The color of petals were compared using CIELAB analysis by spectrophotometer. Five different flowers were chosen for analysis indoors, every flower was tested three times. Fresh petals were kept under −80°C for flavonoid and the total RNA extraction.

### Content measurement of total flavonoids and total anthocyanins

About 0.1 g of samples were used for the determination of total anthocyanin and total flavonoids in each variety. After grinding 0.1 g petals with liquid nitrogen, 1mL extraction solution (0.1% formic acid methanol) was added immediately, and the sample was rapidly and fully mixed with the extraction solution by rapid shock for 30 s. After 24 h extraction at 4°C under dark conditions, add the extraction solution to a constant volume of 3 mL. The absorbance was determined at 524and 325 nm by UV spectrophotometer (Shimadzu, UV2700), and methanol 0.1% formate was used as blank control. The absorbance was determined three times. The standard curve was prepared with 0.1, 0.05, 0.025, and 0.0125 mg⋅mL**^–^**^1^ chlorinated centathrin and rutin standard solution as the standard. The anthocyanin content in the sample was calculated as X = C × B/M. X is the anthocyanin content in the sample (mg.g**^–^**^1^), C is the cornflower pigment content in the extraction liquid (mg⋅mL**^–^**^1^), B is the volume of extraction liquid (mL), and M is the sample mass (g).

### Qualitative and quantitative analysis of flavonoid

Fifteen different colored *L. indica* samples were used for the measurement of (Five cultivars, three biological repetitions each). First, a mixer mill was used to grind the freeze-dried samples (MM 400, Retsch). Then, a solution of 70% methanol in 1.2 mL was required to dissolve 100 mg of flower powder for the entire flavonoid extraction. The sample was rapidly and fully mixed with the extraction solution by rapid shock for 30 s every 30 min for a total of 6 times. After the samples were treated, they were stored overnight at 4°C. Finally, a nylon syringe filter was used to filter the supernatant (SCAA-104, 0.22 μm particle size; ANPEL, Shanghai, China^1^), and remove the sediment by centrifuging for 10 min at 12000 rpm. After sediment was removed and the liquid was clear, flavonoid analysis could be conducted both qualitatively and quantitatively (Fu et al., 2021). The UPLC-ESI-MS/MS equipment (UPLC, SHIMADZU Nexera X2; MS, Applied Biosystems 4500 Q TRAP) was used to examine all extraction solutions. Supplementary Methods 1 and 2 would show the detailed condition of UPLC and ESI-Q TRAP-MS/MS.

Multiple reaction monitoring (MRM) was performed to identify the flavonoids in *L. indica* flowers based on the standard Metabolites Database, which is commercially accessible (Metware Biotechnology Co., Ltd., Wuhan, China). In accordance with secondary spectrum information collected by UPLC based on the system of MRM, signals were collected and analyzed using Analyst 1.6.3. (Metware Biotechnology Co., Ltd., Wuhan, China) to quantify and identify the flavonoids. PCA (principal component analysis) was performed for analyzing the biological repetition condition of fifteen samples (five cultivars, three biological replicates) (Jiang et al., 2020). Significantly differential accumulated flavonoids were analyzed by R package MetaboAnalystR (Chen et al., 2009) based on OPLS_DA (orthogonal partial least squares discriminant analysis) analysis (Thévenot et al., 2015). The standard of screening: VIP ≥ 1 and | log_2_FC| ≥ 1.

### RNA extraction, sequencing, and analysis

Freeze-dried petals were grounded for RNA extraction. A Trizol reagent kit (Invitrogen, Carlsbad, CA, United States) was used to isolate total RNA from petals from five different cultivars. After monitoring total RNA degradation and agarose gel contamination, total RNA purity was determined using a NanoPhotometer^®^ and spectrophotometer (IMPLEN, CA, United States). RNA Nano 6000 Assay Kit for the Bioanalyzer 2100 system was used to quantify total RNA concentration. The integrity of total RNA was detected by Qubit^®^ RNA Assay Kit in the Qubit^®^2.0 Fluorometer. The cDNA library was made by Illumina’s NEBNext^®^ Ultra TM RNA Library Prep Kit and 1 μg of total RNA. Oligo(dT) magnetic beads were used to enrich RNA with polyA and disrupt it randomly. It was used to synthesize the first cDNA, and then used dNTPs as raw materials and mRNA fragments as templates to synthesize the second cDNA. After screening cDNA with AMPure XP Beads, the PCR products were purified again using AMPure XP Beads to obtain the library, which was around 200 bp in length. After examining the quality of the library, all products were sequenced by Illumina.

Fastp v 0.19.3 was used to remove adapters, ploy-N, and low-quality reads from the original data for acquiring clean reads. Trinity was used to assemble the clean reads (v2.11.0) (Grabherr et al., 2011), and the assembled transcripts were clustered to eliminate redundancy using Corset. TransDecoder^2^ was used to identify candidate coding regions within transcript sequences generated by *de novo* RNA-Seq transcript assembly using Trinity. Subsequently, to acquire the annotation of unigenes, As a first step toward obtaining unigene annotation, DIAMOND BLASTX (Buchfink et al., 2015) was performed for functional gene prediction based on the Nr database (NCBI non-redundant), Swiss-prot protein database (Bairoch and Boeckmann, 1991), TrEMBL, KEGG database (Kyoto Encyclopedia of Genes and Genomes) (Kanehisa et al., 2007), as well as the COG (Clusters of Orthologous Groups of proteins) (Tatusov et al., 2000) and GO database (Gene Ontology) (Ashburner et al., 2000). Then, the amino acid sequence was aligned with the Pfam database by HMMER (Finn et al., 2013). The level of gene expression was estimated by RSEM (Li and Dewey, 2011), and the FPKM was calculated based on the gene length of each gene. With the standard of | fold change| ≥ 2 as well as false discovery rate (FDR) < 0.5, DESeq2 (Love et al., 2014) detected DEGs. The enrichment analysis was performed based on the KEGG pathway and GO term. The PCA and sample correlation analysis are shown here in Supplementary Figure 1, and the data from PP2 were eliminated.

Based on iTAK (Zheng et al., 2016), hmmscan (Jin et al., 2013) was used to identify and annotate candidate transcription factors (TFs) that would regulate anthocyanin synthesis. Using DESeq2 to identify differentially expressed TFs using the same standard of pathway genes.

### Interaction network between genes and metabolites

We calculated Pearson’s correlation coefficients (PCC) based on the Metware Cloud^3^ and displayed them using Cytoscape (Shannon et al., 2003) afterward to discover the interconnections between candidate genes, including TFs and pathway genes, and anthocyanin components (v.3.9.1). | PCC| > 0.8 as well as *p*-value 0.05 are the correlation analysis standards.

## Results

### Flower pigment of *Lagerstroemia indica*

The content of total anthocyanins and total flavonoids in petals of different flower colors was measured. RD had the highest total anthocyanins content (868.07 mg⋅mL**^–^**^1^), followed by PP (194.19 mg⋅mL**^–^**^1^), PK (92.63 mg⋅mL**^–^**^1^), VT (70.1 mg⋅mL**^–^**^1^), WH had the lowest anthocyanins content. RD had the highest total flavonoid content, WH was lower than RD, and PK had the lowest (Figure 1B). These results indicated that anthocyanins were the main factors to form the color of crape myrtle, and flavonoids were the main pigment of WH. It should be noted that the color lightness of PP (37.774 ± 1.27) was not significantly different from that of RD (22.188 ± 1.27) (Supplementary Table 1), While the content of anthocyanin in RD was nearly five times that of PP (Figure 1B). Flavonoids are important types of co-pigment, and co-pigment can make the pigment more stable, indicating that flavonoids in PP can better maintain the stability of pigment and make purple more obvious.

To better understand the metabolic changes between five different colored petals, we performed flavonoid analysis based on LC-ESI-MS/MS, and five typical cultivars were selected for this study. PCA was used to measure the trend of metabolic separation between all groups. The result of PCA showed that all samples were separated into five groups on the PC1 × PC2 score plot (Supplementary Figure 1). The results showed that the biological repeatability among the five groups was good, and intragroup correlations were high.

A total of 299 compounds were identified in total across all samples (Figure 1C). All metabolites can be categorized into 12 classes further, including tannins (87), flavonols (79), flavones (44), flavanones (24), anthocyanidins (14), isoflavones (11), flavanols (11), flavanonols (10), chalcones (eight), flavonoid carbonoside (eight), dihydroisoflavones (two), and proanthocyanidins (one).

From PCA of all samples, significant differences were shown between groups. OPLS_DA was performed for five groups of samples to analyze differential expression. Non-repetitive comparisons had Q2 values greater than 0.9, and the *p*-values of models were all below the threshold of 0.05, which indicated that models built by each group were capable of making accurate predictions (Supplementary Figure 2).

Differential accumulated metabolites were screened based on OPLS_DA model results (Figure 1D). There were 191 DEMs in PK vs. PP (99 down-regulated, 92 up-regulated), 192 DEMs in RD vs. PK (130 down-regulated, 62 up-regulated), 135 DEMs in RD vs. PP (129 down-regulated, 6 up-regulated), 170 DEMs in VT vs. PK (71 down-regulated, 99 up-regulated), 113 DEMs in VT vs. PP (40 down-regulated, 73 up-regulated), 162 DEMs in VT vs. RD (14 down-regulated, 148 up-regulated), 191 DEMs in WH vs. PK (61 down-regulated, 130 up-regulated), 163 DEMs in WH vs. PP (63 down-regulated, 100 up-regulated), 176 DEMs in WH vs. RD (23 down-regulated, 153 up-regulated), and 168 DEMs in WH vs. VT (77 down-regulated, 91 up-regulated).

### Anthocyanins among the petal of *Lagerstroemia indica* cultivars

**FIGURE 2:**
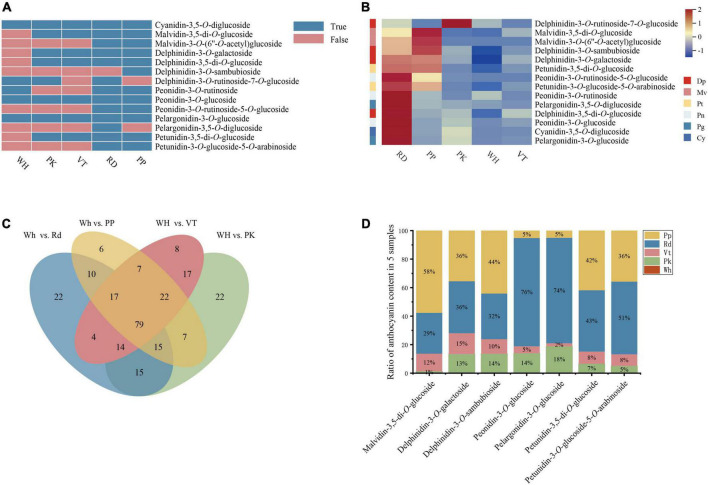
Composition and content of anthocyanin among five *L. indica* petals. **(A)** Type of anthocyanin detected in *L. indica* petals. **(B)** Heatmap of all anthocyanins. **(C)** Venn analysis among WH vs. RD, WH Vs. PP, WH vs. VT, and WH vs. PK. **(D)** The ratio of relative content of overlapped DAAs in five samples.

Anthocyanins were significant contributors to color formation. To know the certain type of anthocyanin that have a great influence on the color diversity of *L. indica*, the detected anthocyanins were analyzed further. 14 types of anthocyanin were detected from five cultivars (Figure 2A). There were four delphinidin derivatives, two malvidin derivatives, three peonidin derivatives, two pelargonidin derivatives, two petunidin derivatives, and one cyanidin derivative. From the heatmap of all detected anthocyanins (Figure 2B), derivatives of peonidin, pelargonidin, and Cyanidin had higher accumulation levels in RD and PK than in other samples. These pigments can form red color. While accumulation level of delphinidin derivatives and malvidin derivatives was higher in PP and VT, and those two types of anthocyanin had high relation to purple-blue-hued flower color. Besides, malvidin-3-*O*-(6′′-*O*-acetyl) glucoside and petunidin-3-*O*-glucoside-5-*O*-arabinoside were only found in PP and RD. Pelargonidin-3,5-*O*-diglucoside and delphinidin-3-*O*-sambubioside were specifically accumulated in RD and PP respectively. Delphinidin-3-*O*-rutinoside-7-*O*-glucoside was only detected in PK, RD, and WH, and it was accumulated higher in PK than in RD and WH. Malvidin-3-*O*-(6′′-*O*-acetyl) glucoside, pelargonidin-3,5-*O*-diglucoside, delphinidin-3-*O*-sambubioside, and delphinidin-3-*O*-rutinoside-7-*O*-glucoside might have an important role in forming the red color, purple color, and pink color.

To know the formation of petal colors, a Venn diagram was built with four comparisons (WH vs. RD, WH vs. PK, WH vs. PP, WH vs. VT), and there were 79 overlapped differential accumulated metabolites (Figure 2C). Among these, there were seven differential accumulated anthocyanins (DAAs) up-regulated in PK, RD, PP, and VT compared with WH, respectively, including delphinidin-3-*O*-galactoside, delphinidin-3-*O*-sambubioside, petunidin-3-*O*-glucoside-5-*O*-arabinoside, petunidin-3,5-di-*O*-glucoside, malvidin-3,5-di-*O*-glucoside, peonidin-3-*O*-glucoside, pelargonidin-3-*O*-glucoside. Compared with the fold change value of overlapped differential accumulated metabolites between four colored sample comparisons, malvidin-3,5-di-*O*-glucoside had a great value (Supplementary Table 2), which indicated that it might be the key pigment that influences color diversity in crape myrtle flowers (Figure 2D).

### Analysis of the RNA-seq data

Flower petals of *L. indica* were used to perform RNA-seq for potential molecular mechanism analysis. After filtering low-quality reads from the raw data, fifteen samples (5 cultivars and three biological replicates each) were used to establish libraries. A total of 99.54 Gb clean reads were generated, and 6.09−8.89 Gb for each cultivar was obtained by sequence The proportion of both Q20 and Q30 bases in each library was greater than 90% respectively. And the GC content was ranging from 49.1% to 52.02% (Supplementary Table 3). 149,235 Transcripts were assembled by Trinity as a reference sequence. After eliminating redundancy, 141,673 unigenes were generated with an N50 length of 1,701 bp, an N90 length of 430 bp, and the mean length of 1,045 bp (Supplementary Table 4). The length of unigene around 300−400 bp (21,341, 15.06%) was the most abundant followed by 200−300 bp (21,211, 14.97%), 400−500 bp (14,348, 10.13%), over 2,000 bp (20349, 14.36%). While the unigene with a length of 1,800−1,900 bp accounted for the smallest proportion (Supplementary Table 5). To better know the gene function, all unigenes were compared to seven functional database; 141,672 unigenes were annotated. Among them, NR database and Trembl database annotated the most unigenes, and the ratio of annotation was 61.18% (86,670) and 61.12% (86,585) respectively. The number of unigenes compared to GO database was 71,292 (50.32%) after the NR database and the Trembl database. In addition, the number of annotations in the KEGG database, SwissProt database, and Pfam database was 62,812 (44.34%), 62,732 (44.28%), and 62,036 (43.79%), respectively (Supplementary Table 6). Besides, the ratio successfully annotated to the KOG database was the least (52,140, 36.8%). 70.85% of unigenes of *L. indica* shared the highest similarity (70.85%) with *Punica granatum* based on the NR database (Supplementary Figure 3). The results of GO annotation showed that all unigenes were successfully categorized into three major categories, including biological process, cellular component, and molecular function. And GO functions involved 60 subcategories. Among them, most of unigenes were enriched in the subcategories cell (53,405, 74.91%), cell part (53,282, 74.74%), cellular process (45,240, 63.46%), binding (42,942, 60.23%), organelle (40,749, 57.16%), and metabolic process (38,792, 54.41%; Supplementary Figure 4). In addition, the KOG database was used for orthologous protein annotation. As a result, 25 KOG functional categories were annotated, general function prediction only enriched the majority of genes, and the number of it is 9,948. The number of genes annotated in posttranslational modification, protein turnover, chaperones followed subsequently (Supplementary Figure 5).

### Differentially expressed genes between different colored flowers

**FIGURE 3:**
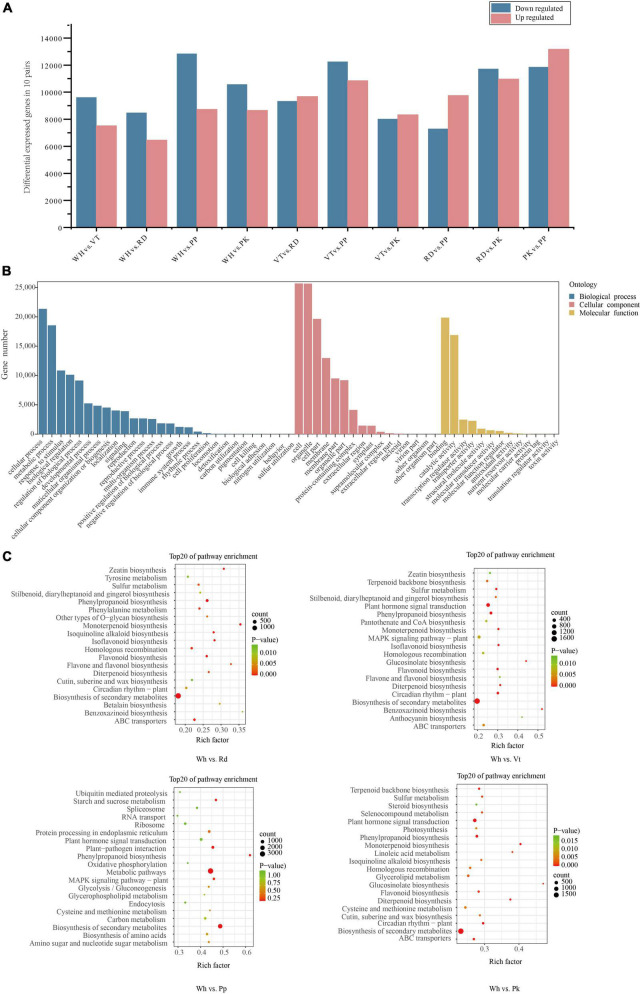
Statistical analysis of DEGs, GO, and KEGG enrichment analysis of all DEGs. **(A)** Number of DEGs among all comparison units. **(B)** Go enrichment of all DEGs. **(C)** KEGG enrichment analysis among WH vs. RD, WH Vs. PP, WH vs. VT, and WH vs. PK. The top 20 enriched pathways are shown.

By analyzing transcripts obtained from the transcriptome, DEGs in different colored samples were identified based on their FPKM values. There were 17,131 (WH vs. VT), 14,940 (WH vs. RD), 21,570 (WH vs. PP), 19,223 (WH vs. PK), 19,011 (VT vs. RD), 23,096 (VT vs. PP), 16,348 (VT vs. PK), 17,038 (RD vs. PP), 22,691 (RD vs. PK), and 25,028 (RD vs. PP) DEGs in 10 comparison groups, respectively (Figure 3A). To know the gene function of DEGs, gene functional annotation was performed. From GO annotation of all DEGs. A total of 33,534 DEGs were distributed into 58 terms, including 13 molecular functions, 16 cellular components, 28 biological processes, and 55.31% in biological processes. The greatest abundance terms were cellular process, metabolic process, response to stimulus, and biological regulation. Under cellular component, cell, cell part, organelle, and membrane were the most abundant terms. Binding and catalytic activity contained the most DEGs within the biological process (Figure 3B).

To further investigate the metabolic pathway of the DEGs, KEGG pathway enrichment of DEGs between five cultivars, including WH vs. VT, WH vs. RD, WH vs. PP, WH vs. PK, VT vs. RD, VT vs. PP, VT vs. PK, RD vs. PP, RD vs. PK, and PP vs. PK, respectively. The top 20 enriched pathways were shown in all comparisons (Figure 3C and Supplementary Figure 6). From the results, the pathway of biosynthesis of secondary metabolites, metabolic pathways, phenylalanine metabolism, and flavonoid biosynthesis, which were connected to anthocyanin synthesis, were enriched in the VT, PP, RD, and PK.

### The differential expressed structural genes regulating the flavonoid biological synthesis

**FIGURE 4:**
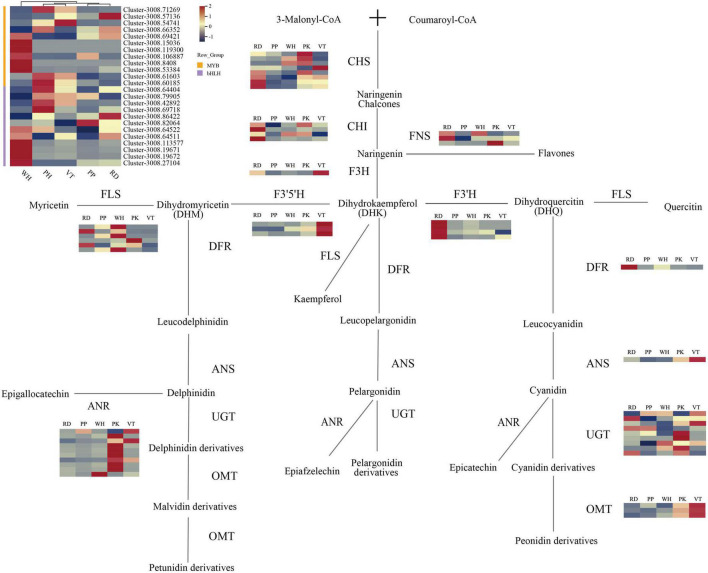
Anthocyanin biosynthesis pathway in *L. indica* flowers. Each colored cell represents the average FPKM value standardized by the Z-Score of each gene.

By analyzing KEGG relationships and gene annotations, DEGs related to the biological synthesis of flavonoids were identified. As a result, 53 DEGs on the flavonoid pathway were discovered, including eight *CHS*, four *CHI*, one *F3H*, three *F3*′*5*′*H*, four *F3*′ *H*, and one *DFR*, one *ANS*, *three OMT*, nine *UGT*, six *FLS*, three *FNS*, and 10 *ANR*. All these 53 DEGs were identified in 10 unrepetitive comparison pairs of PK, RD, PP, VT, and WH. From the heatmap (Figure 4), *CHS* and *CHI* gene expression of PP was relatively lower than others. For the *F3H* gene, the expression of VT was higher than PK and RD, while the upstream genes (*CHS* and *CHI*) of the PK and RD had higher expression. *FNS* genes of PK and RD had higher expression. Enzyme FNS and F3H catalyzed the same substrate naringenin, which indicated that when naringenin was transformed into Dihydrokaempferol (DHK) and flavones, more substrate flowed to the synthesis of flavones. At the same time, the *FNS* gene of the WH sample was also highly expressed, which indicated that the flavonoid accumulation of the WH sample was higher than that of the other samples.

When it comes to the expression of the *F3*′*5*′*H* and *F3*′*H* genes in five cultivars, there were some interesting findings. The expression of the *F3*′*5*′*H* gene in VT was much higher than in other samples, but the expression of the *F3*′*H* gene was relatively low. *F3*′*5*′*H* gene and *F3′H* gene co-catalyzed the substrate DHK, indicating that there was competition for substrate allocation in VT, and ultimately more substrate flows to the synthesis of dihydromyricetin (DHM). This tributary led to the synthesis of delphinidin derivatives, malvidin derivatives, and peonidin derivatives. From the metabolome analysis of VT, VT contained four such compounds, accounting for 57.14% of all anthocyanin types. The results of gene expression in VT can also be consistent with the metabolome analysis. The expression levels of the *F3*′*H* gene in RD were higher than in others, which indicated that the substrate DHK flowed more toward the synthesis of dihydroquercetin (DHQ) than toward the cyanidin and peonidin derivatives. The metabolome’s findings corroborated these findings. Although the expression of pathway genes was low in the PP, it indicated that pathway genes of PP may be more efficient when coding relevant protein. However, the flower color of PP was darker than PK and VT, and the total content of anthocyanin was also high. In contrast to the RD of the same dark-colored flowers, the expression level of the gene was low. It is, therefore, possible that the active period for anthocyanin accumulation in PP may not be during the full bloom period.

The expression levels of *FNS* genes and *FLS* genes were higher in white samples, while the expression levels of structural genes in the anthocyanin synthesis pathway were lower. It indicated that the final product of the catalytic substrate did not ultimately flow toward the synthesis of anthocyanins but more toward the synthesis of flavanones. *DFR* and *ANS* were downstream genes of anthocyanin synthesis. The expression level of *DFR* in PK was low, and *FLS* expressed highly in PK, which meant more DHQ flowed to the flavone synthesis. *OMT* genes and *UGT* genes were responsible for the final modification of anthocyanidins. It could be seen from the heatmap that all colored samples had high expression levels of *UGT*. The expression of *OMT* genes in VT samples was higher than in other samples, followed by PK. *OMT* genes could be involved in the methylation of anthocyanins. There were three kinds of anthocyanins in PK and VT that need to undergo multiple methylations including malvidin-3,5-di-*O*-glucoside, peonidin-3-*O*-glucoside, and petunidin-3,5-di-*O*-glucoside. The expression of *OMT* genes was lower in PP and RD than in PK and VT, but the seven anthocyanins in PP and RD were required to undergo multiple methylations.

### Identification of candidate transcription factors and correlation network of key differentially expressed genes and differential accumulated anthocyanins

**FIGURE 5:**
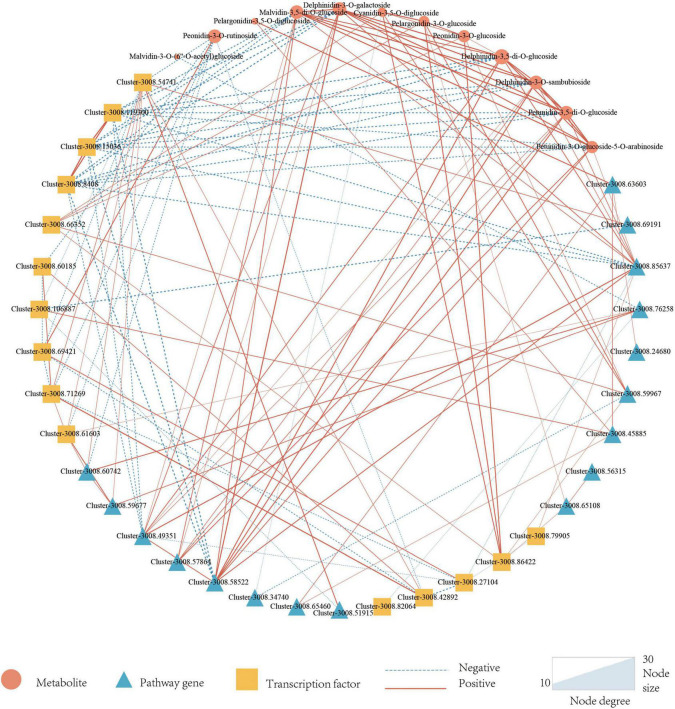
Correlation network of candidate metabolites, Pathway genes, and Transcription factors. Triangles represent metabolites, circles represent pathway genes, squares represent transcription factors, dotted lines represent negative associations, and solid lines represent positive associations. Metabolites are set as target nodes, which are determined by the number of genes associated with them.

In addition to pathway genes, TFs also played an important role in regulating the synthesis of plant anthocyanins. There were three main transcription factors involved in the regulation of anthocyanin synthesis including MYB, bHLH, and WD40. A total of 5317 TFs were annotated in this study, of which 184 (3.46%) were annotated for MYB, 257 (4.83%) for bHLH, and WD40 was not annotated. Differential expression analysis of the annotated TFs was performed, and finally, 11 MYBs and 5 bHLHs that might be involved in the regulation of anthocyanin synthesis were screened. To better know the relationship between anthocyanins, DEGs, and candidate TFs, a correlation network map was constructed by 14 anthocyanins, 34 structural genes, and 24 TFs. It could be seen from Figure 5 that there were 22 genes directly related to anthocyanins, including 11 TFs and 10 structural genes. 11 TFs included nine MYBs and two bHLHs, 10 pathway genes included one *CHS* gene, two *CHI* genes, one *F3*′*H* gene, one *F3*′*5*′*H* gene, one *OMT* gene, and four *UGT* genes.

As a result, only 12 anthocyanins were strongly correlated with candidate structural genes and TFs, and more genes were correlated with delphinidin-3-*O*-galactoside, delphinidin-3-*O*-sambubioside, malvidin-3,5-di-*O*-glucoside, peonidin-3-*O*-rutinoside, petunidin-3,5-di-*O*-glucoside. Malvidin-3,5-di-*O*-glucoside, delphinidin-3-*O*-galactoside, delphinidin-3-*O*-sambubioside, and petunidin-3,5-di-*O*-glucoside are derivatives of malvidin, delphinidin, and petunidin, which all belonged to the final products of the lower tributaries catalyzed by *F3′5′H* enzyme. Among malvidin-3,5-di-*O*-glucoside related genes, three *MYB* genes were negatively correlated with malvidin-3,5-di-*O*-glucoside, and four pathway genes were positively correlated with malvidin-3,5-di-*O*-glucoside (one *F3*′*5*′*H* gene and three *OMT* genes). The correlation of transcription factor MYB was generally higher than that of pathway genes, and delphinidin-3-*O*-sambubioside, delphinidin-3-*O*-galactoside, and Petunidin-3,5-di-*O*-glucoside were consistent with that of malvidin-3,5-di-*O*-glucoside. In petunidin-3,5-di-*O*-glucoside and petunidin-3-*O*-glucoside-5-*O*-arabinoside, three identical MYB TFs were negatively correlated with them, and three pathway genes were positively correlated with them. The three structural genes included two *UGT* genes and one *F3*′*5*′*H* gene.

Six of the seven genes with a high correlation with peonidin-3-*O*-rutinoside were TFs and only one pathway gene. In addition, only one TF was positively correlated with the seven genes, and the value of | PCC| was higher than the other six genes. Four genes related to cyanidin-3,5-di-*O*-glucoside contained two TFs and two pathway genes, all those two TFs had a positive correlation with cyanidin-3,5-di-*O*-glucoside. One of the two pathway genes is positively correlated and the other is negatively correlated. All three genes related to peonidin-3-*O*-glucoside were positively correlated, including two TFs and one pathway gene. All genes associated with pelargonidin-3-*O*-glucoside and pelargonidin-3,5-*O*-diglucoside were positively correlated. Four genes (two transcription factors and two pathway genes) were related to pelargonidin-3-*O*-glucoside, and two structural genes were related to pelargonidin-3,5-*o*-diglucoside.

Among all TFs highly correlated with anthocyanins, most were negatively correlated, including seven MYBs and one bHLH. Only three TFs were positively correlated, including two MYBs and one bHLH. Most of the structural genes were positively correlated with anthocyanins, and only two were negatively correlated, namely, one *UGT* and one *OMT*. Structural genes are responsible for anthocyanin skeleton synthesis f transcription factors are responsible for the regulation of structural genes. Therefore, it is reasonable that most structural genes are positively correlated while most transcription factors are negatively correlated.

The correlation coefficients of the *F3*′*5*′*H* gene (Cluster-3008.85637) and the *F3*′*H* gene (Cluster-3008.63603) were significantly higher than upstream genes *CHS* (Cluster-3008.59967) and *CHI* (Cluster, 3008.65108-3008.45885) in anthocyanin synthesis pathway. It can be concluded that the *F3*′*5*′*H* gene and *F3*′*H* gene play a more important role in anthocyanin synthesis.

## Discussion

### Malvidin-3,5-di-*O*-glucoside may be responsible for the color diversity in *Lagerstroemia indica* flowers

Anthocyanins take the main responsibility for the coloration of petals of plants (Zhuang et al., 2019). Like the flowers of other species, anthocyanin was also enriched in the petals of *L. indica*. There were 14 different kinds of anthocyanin identified in five cultivars, and the level of accumulation of anthocyanin was higher in colored petals than in white petals. In addition, the color of the flower is mainly decided by the type and content of the pigment in the plant tissue. In this study, five types only existed in specific-colored samples, these pigments might play an important role in establishing the color. And seven DAAs were identified among WH vs. RD, WH vs. PP, WH vs. PK, and WH vs. VT, which might be the contribution to the different petal colors. Analyzing the relative content of these seven anthocyanins in different samples, the relative content ratio of malvidin-3,5-di-*O*-glucoside varied greatly in different samples. In addition, from the correlation network, malvidin-3,5-di-*O*-glucoside had a high correlation with candidate genes involved in the synthesis of flavonoids. This finding could support the results of Zhang et al. (2008), who pointed out that malvidin-3-*O*-glucoside is one of the main pigments in crape myrtle flowers. Malvidin was mainly connected with the purple flowers. Malvidin-3,5-di-*O*-glucoside is detected in the flower of *Salvia miltiorrhiza* and *Glycine soja* and was responsible for the formation of the purple flowers (Takahashi et al., 2010; Jiang et al., 2020). In this study, malvidin-3,5-di-*O*-glucoside was presented in all colored flowers and was highly accumulated in PP, and the level of accumulation in VT is higher than in PK. While RD and PK also contained malvidin-3,5-di-*O*-glucoside, it indicated that the final formation of red color and pink color in RD and PK needs the participation of other kinds of anthocyanins. In the study of Zhang et al. (2008), the content of delphinidin-3-*O*-glucoside was mentioned to be one of the key pigments for breeding the blue-colored crape myrtle flower. In previous studies, delphinidin derivatives were considered the key pigment of most blue flowers (Qi et al., 2013). In this study, four delphinidin derivatives (delphinin-3-*O*-galactoside, delphinidin-3-*O*-sambubioside, delphinidin-3,5-*O*-diglucoside, and delphinidin-3-*O*-rutinoside-7-*O*-glucoside) were found. Malvidin derivatives were the downstream production of delphinidin derivatives, delphinidin derivatives could be transformed into malvidin derivatives through the modification by the gene *OMT*. It indicated that there was a competition between the synthesis of delphinidin and malvidin that might hinder the accumulation of delphinidins for blue flowers. While the true content of every pigment in each sample would not be known through relative content detection. Further study is needed to know the main pigment of each sample and analyze the absolute quantification analysis of each colored sample.

### The expression of *F3′5′H* and *F3′H* may have a great influence on color formation

The biosynthesis pathway of anthocyanin was reported in Holton and Cornish (1995), and the function of key genes in the anthocyanin pathway was explored in recent studies. As reported, the expression of *F3*′*5*′*H* and *F3*′*H* is the key that can control the carbon flux to different branches in the pathway of anthocyanin synthesis (Shimada et al., 2001; Jeong et al., 2006).

The pigment synthesis of red or purple/blue flowers was controlled by *F3*′*H* and *F3*′*5*′*H*, respectively. Malvidin was replaced by peonidin as the primary anthocyanin in petunia transgenics overexpressing grape *VvF3*′*5*′*H* in the petunia mutant *ht1*, and the flower color turned to purple (Bogs et al., 2005). The fruit skin of *Lycium barbarum* appeared red because of the lack of expression of *F3*′*5*′*H* (Zeng et al., 2014). Suppressing the expression of *F3*′*5*′*H* led to the decrease of delphinidin derivatives and resulted in the red flower in the gentian (Nakatsuka et al., 2008). Therefore, the different transcriptional levels of *F3*′*H* and *F3*′*5*′*H* would decide the type and ratio of final pigments in flowers and ultimately form different flower colors. In this study, the levels of *F3*′*H* and *F3*′*5*′*H* expressions in five colored samples (white, pink, red, violet, and purple) were different. RD (red flowers) had a high level of *F3*′*H* expression, and *F3*′*5*′*H* was expressed highly in VT (violet flowers). And this would result in the color diversity of *L. indica.*

In addition, *F3′5′H* and *F3′H* as the center of the anthocyanin synthesis branch had different preferences and catalytic activity for the substrate (Olsen et al., 2010; Wang et al., 2014; Miyahara et al., 2016). And *F3′5′H* and *F3′H* would compete for common substrates (DHK), so the different preferences of *F3′H* and *F3′5′H* for substrates could ultimately affect the direction of substrate flow to a certain extent and influence the diversity of pigments in plants and lead to the different color formation. Transforming *F3*′*5*′*H* of prairie gentian to pink petunia, enzyme *F3′H* competed with the DHK with enzyme *F3′5′H*, and the color of the flowers turned to purplish red. In this experiment, we noticed *F3′H* had expression in purplish flowers and *F3′5′H* was expressed also in reddish flowers. It meant *F3′H* and *F3′5′H* would have a high possibility of competing for the same substrate in different samples, which could affect the ratio of substrates assigned to the two tributaries, thus affecting the formation of the final color of flowers. Besides, *F3′5′H* would control the synthesis of the key anthocyanin malvidin-3,5-di-*O*-glucoside. Thus, we considered *F3*′*H* and *F3*′*5*′*H* as the key to decoding the diverse color in *L. indica*.

For TFs shown in the correlation network diagram (Figure 5), 11 transcription factors were shown to be related to the synthesis of anthocyanins, among which only one of the three MYBs was negatively correlated with the content of malvidin-3,5-di-*O*-glucoside. Some studies have reported that the MYB transcription factor in *L. indica* had a higher preference for the regulation of anthocyanins (Yu et al., 2021). However, how MYB regulated the expression of structural genes and thus influenced the synthesis of anthocyanins is still unclear. In future research, we will focus on the study of the expression of the *F3*′*5*′*H* gene and *F3*′*H* gene in the crape myrtle, as well as the condition of interactions between MYB transcription factors and structural genes in crape myrtle.

Taken together, our study showed that malvidin-3,5-di-*O*-glucoside might be the key pigments for the color diversity of crape myrtle. *F3*′*H* and *F3*′*5H* are two key genes in the anthocyanin synthesis pathway, that might have a great influence on the synthesis of anthocyanins and the final color formation of flowers. 11 TFs which might have a great influence on the key pigment synthesis were identified, including nine MYBs and two bHLH.

## Data availability statement

The original contributions presented in this study are publicly available. This data can be found here: NCBI, BioProject PRJNA818829 and BioSample SAMN26884420.

## Author contributions

SH contributed to the conceptualization, performed the methodology, investigated and validated the data, wrote the original draft, and visualized the data. JW contributed to the conceptualization, performed the methodology, validated and visualized the data, and wrote the original draft. QW contributed to the conceptualization, performed the methodology, investigated and visualized the data, and wrote the original draft. YZ and GZ performed the methodology and validated the data. QM performed the methodology and investigated the data. ZW contributed to the conceptualization, performed the methodology, and wrote, reviewed, and edited the manuscript. JM contributed to the conceptualization, performed the methodology, and wrote, reviewed, and edited the manuscript. CG contributed to the conceptualization, performed the methodology, carried out the resources, supervised the data, and carried out the funding acquisition. All authors contributed to the article and approved the submitted version.

## Footnotes

1
http://www.anpel.com.cn/

2
https://github.com/TransDecoder/

3
https://cloud.metware.cn/#/tools/tool-list
