# Supplementary material for: Decoding the formation of diverse petal colors of Lagerstroemia indica by integrating the data from transcriptome and metabolome
Source: Front Plant Sci. 2022 Sep 7;13:970023. doi: 10.3389/fpls.2022.970023 (PMC9490092; doi:10.3389/fpls.2022.970023)
Supplement: Supplementary file 1 [file Data_Sheet_1.docx]

Table S1 CIELab value of five samples, L for brightness, a for red-green, b for yellow-blue, c for saturation, h for hue.

| Sample | L | a | b | C | h |
| --- | --- | --- | --- | --- | --- |
| WH | 92.51±0.42 | 0.055±0.26 | 5.0075±0.27 | 5.015±0.27 | 89.5425±2.87 |
| PK | 50.248±1.48 | 37.278±2.43 | -4.016±1.33 | 37.526±2.30 | 354.128±2.40 |
| RD | 22.188±1.27 | 27.834±1.80 | 8.592±0.72 | 29.134±1.9 | 17.016±0.57 |
| PP | 37.774±1.27 | 22.062±1.36 | -17.976±0.91 | 28.494±1.18 | 320.818±2.25 |
| VT | 46.9375±1.41 | 12.7075±3.01 | -13.13±3.014 | 18.2725±4.25 | 314.0425±0.77 |

Table S2 The fold change of common differential accumulated anthocyanins between four colored samples comparisons.

| Metabolites | PK Vs. RD | PK Vs. PP | PK Vs. VT | RD Vs. PP | RD Vs. VT | PP Vs. VT |
| --- | --- | --- | --- | --- | --- | --- |
| Pelargonidin-3-O-glucoside | 4.03 | 0.27 | 0.11 | 0.07 | 0.03 | 0.41 |
| Peonidin-3-O-glucoside | 5.59 | 0.39 | 0.36 | 0.07 | 0.06 | 0.00 |
| Delphinin-3-O-galactoside | 2.71 | 2.64 | 0.00 | 0.00 | 0.40 | 0.41 |
| Pelargonidin-3,5-O-diglucoside | 0.00 | 0.00 | 0.00 | 0.12 | 0.00 | 0.00 |
| Delphinidin-3-O-sambubioside | 2.36 | 3.26 | 0.00 | 0.00 | 0.32 | 0.23 |
| Cyanidin-3,5-di-O-glucoside | 3.60 | 0.34 | 0.09 | 0.10 | 0.03 | 0.27 |
| Petunidin-3-O-glucoside-5-O-arabinoside | 9.99 | 7.02 | 0.00 | 0.00 | 0.16 | 0.23 |
| Delphinidin-3,5-O-diglucoside | 4.13 | 0.00 | 0.00 | 0.00 | 0.27 | 0.00 |
| Petunidin-3,5-di-O-glucoside | 6.48 | 6.29 | 0.00 | 0.00 | 0.20 | 0.20 |
| Malvidin-3,5-di-O-glucoside | 22.28 | 44.93 | 9.65 | 2.02 | 0.43 | 0.21 |
| Peonidin-3-O-rutinoside-5-O-glucoside | 0.00 | 0.00 | 0.00 | 0.47 | 0.00 | 0.00 |
| Delphinidin-3-O-rutinoside-7-O-glucoside | 0.27 | 0.01 | 0.02 | 0.05 | 0.07 | 0.00 |

Table S3 Statistics of RNA-seq. Q30 (%) indicates the percentage of bases with the quality value ≥ 30,GC Content(%) indicates the sum of the number of G and C in high-quality reads as a percentage of the number of bases

| Sample | Raw Reads | Clean Reads | Clean Base(G) | Error Rate(%) | Q20(%) | Q30(%) | GC Content(%) |
| --- | --- | --- | --- | --- | --- | --- | --- |
| Pink1 | 48527496 | 43434482 | 6.52 | 0.02 | 98.35 | 95.03 | 51.02 |
| Pink2 | 47140146 | 43222766 | 6.48 | 0.03 | 97.69 | 93.48 | 51.1 |
| Pink3 | 47139442 | 41617516 | 6.24 | 0.02 | 98.13 | 94.48 | 51.35 |
| Purple1 | 48629592 | 41050738 | 6.16 | 0.02 | 98.42 | 95.18 | 50.81 |
| Purple2 | 49998942 | 41821716 | 6.27 | 0.02 | 98.11 | 94.43 | 50.25 |
| Purple3 | 48778326 | 46544998 | 6.98 | 0.03 | 97.66 | 93.34 | 49.1 |
| Red1 | 61402468 | 59264726 | 8.89 | 0.03 | 97.87 | 93.96 | 50.35 |
| Red2 | 47730570 | 40613418 | 6.09 | 0.02 | 98.06 | 94.34 | 51.26 |
| Red3 | 48604926 | 45210062 | 6.78 | 0.03 | 97.65 | 93.37 | 50.79 |
| Violet1 | 48792412 | 46193706 | 6.93 | 0.03 | 97.75 | 93.63 | 51.17 |
| Violet2 | 47410676 | 41301382 | 6.2 | 0.02 | 98.37 | 95.08 | 51.57 |
| Violet3 | 49422516 | 43449212 | 6.52 | 0.02 | 98.38 | 95.07 | 52.02 |
| White1 | 45552000 | 41871406 | 6.28 | 0.03 | 97.62 | 93.36 | 51.95 |
| White2 | 48281622 | 40428260 | 6.06 | 0.02 | 98.11 | 94.46 | 50.73 |

Table S4 The results of transcripts assembled. N50/N90 indicates arrange the transcripts assembled in descending order of length, ranging from the Cumulative length of the transcripts to the length of the assembled transcripts that are not less than 50%/90% of the total length.

| Type | Number | Mean Length | N50 | N90 | Total Bases |
| --- | --- | --- | --- | --- | --- |
| Transcript | 149235 | 1004 | 1679 | 406 | 1.5E+08 |
| Unigene | 141673 | 1045 | 1701 | 430 | 1.48E+08 |

Table S5 length distribution of transcripts and unigenes.

| distribution | Transcript | Unigene |
| --- | --- | --- |
| 200~300 | 28462 | 21211 |
| 300~400 | 21604 | 21341 |
| 400~500 | 14373 | 14348 |
| 500~600 | 10276 | 10259 |
| 600~700 | 8126 | 8124 |
| 700~800 | 6425 | 6423 |
| 800~900 | 5275 | 5274 |
| 900~1000 | 4733 | 4733 |
| 1000~1100 | 4116 | 4116 |
| 1100~1200 | 3827 | 3827 |
| 1200~1300 | 3524 | 3524 |
| 1300~1400 | 3226 | 3226 |
| 1400~1500 | 2875 | 2875 |
| 1500~1600 | 2704 | 2704 |
| 1600~1700 | 2544 | 2544 |
| 1700~1800 | 2453 | 2453 |
| 1800~1900 | 2226 | 2226 |
| 1900~2000 | 2116 | 2116 |
| >=2000 | 20350 | 20349 |

Table S6 Information on unigene gene annotation

| Database | Number of Genes | Percentage (%) |
| --- | --- | --- |
| KEGG | 62812 | 44.34 |
| NR | 86670 | 61.18 |
| SwissProt | 62732 | 44.28 |
| Trembl | 86585 | 61.12 |
| KOG | 52140 | 36.80 |
| GO | 71292 | 50.32 |
| Pfam | 62036 | 43.79 |
| Annotated in at least one Database | 91342 | 64.47 |
| Total Unigenes | 141673 | 100.00 |
