# Supplementary material for: Decoding the formation of diverse petal colors of Lagerstroemia indica by integrating the data from transcriptome and metabolome
Source: Front Plant Sci. 2022 Sep 7;13:970023. doi: 10.3389/fpls.2022.970023 (PMC9490092; doi:10.3389/fpls.2022.970023)
Supplement: Supplementary file 2 [file Data_Sheet_2.docx]

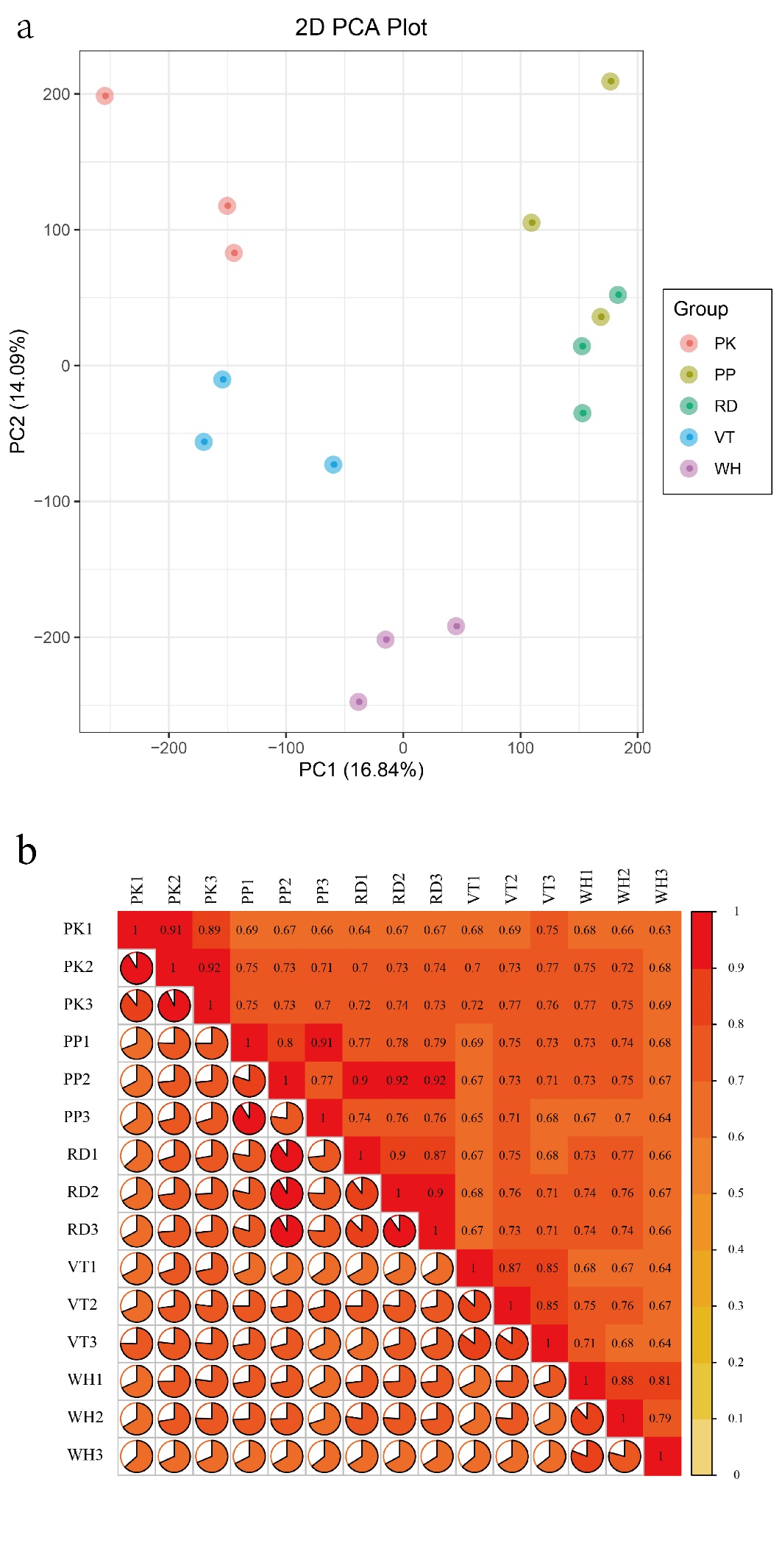


Figure S1 PCA and correlation analysis of all samples (a) Principal component analysis (PCA) of unigene. (b) Sample correlation analysis.


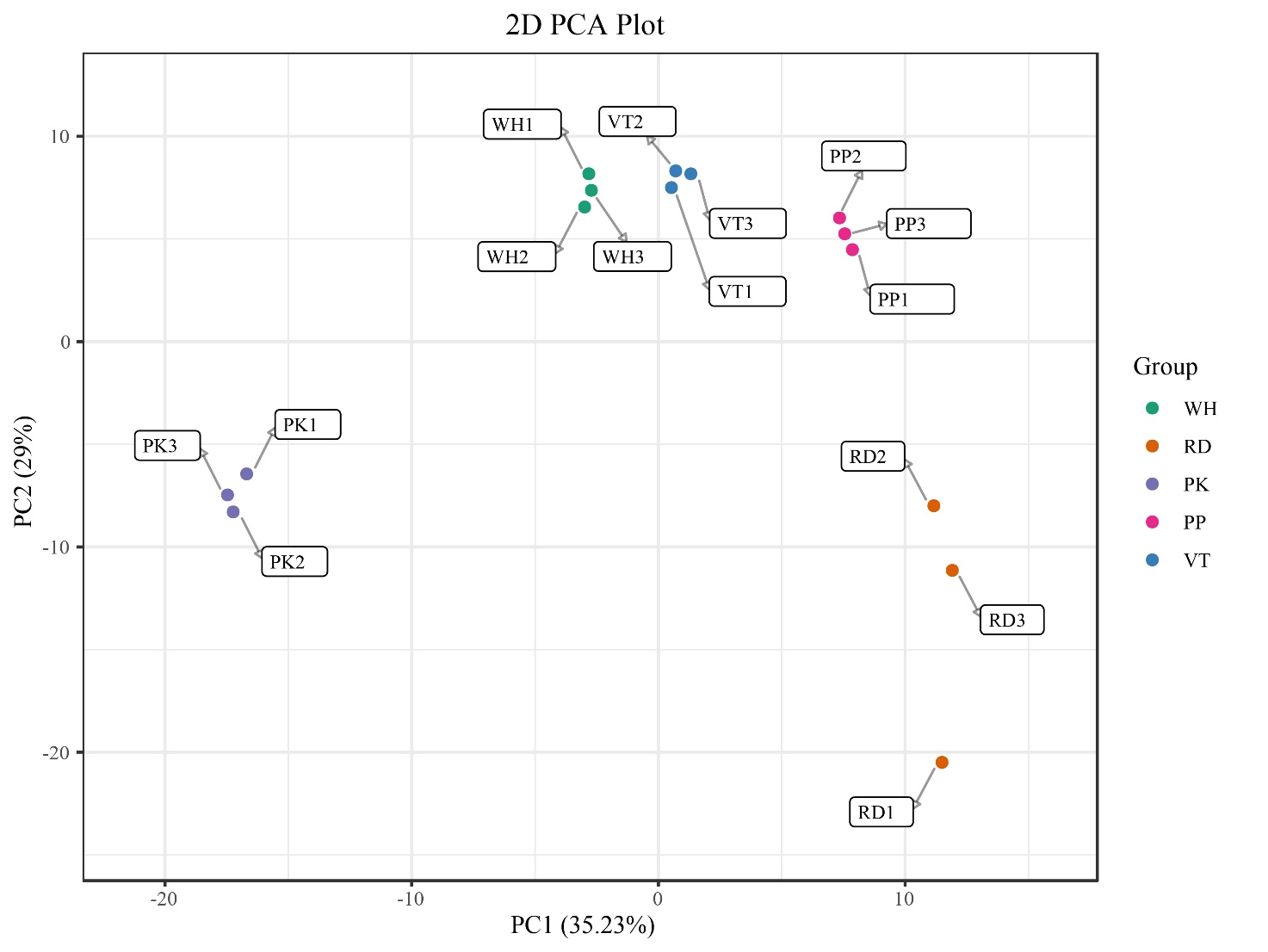


Figure S2 Principal component analysis (PCA). The PCA was derived from the relative content of all detected flavonoid metabolites.


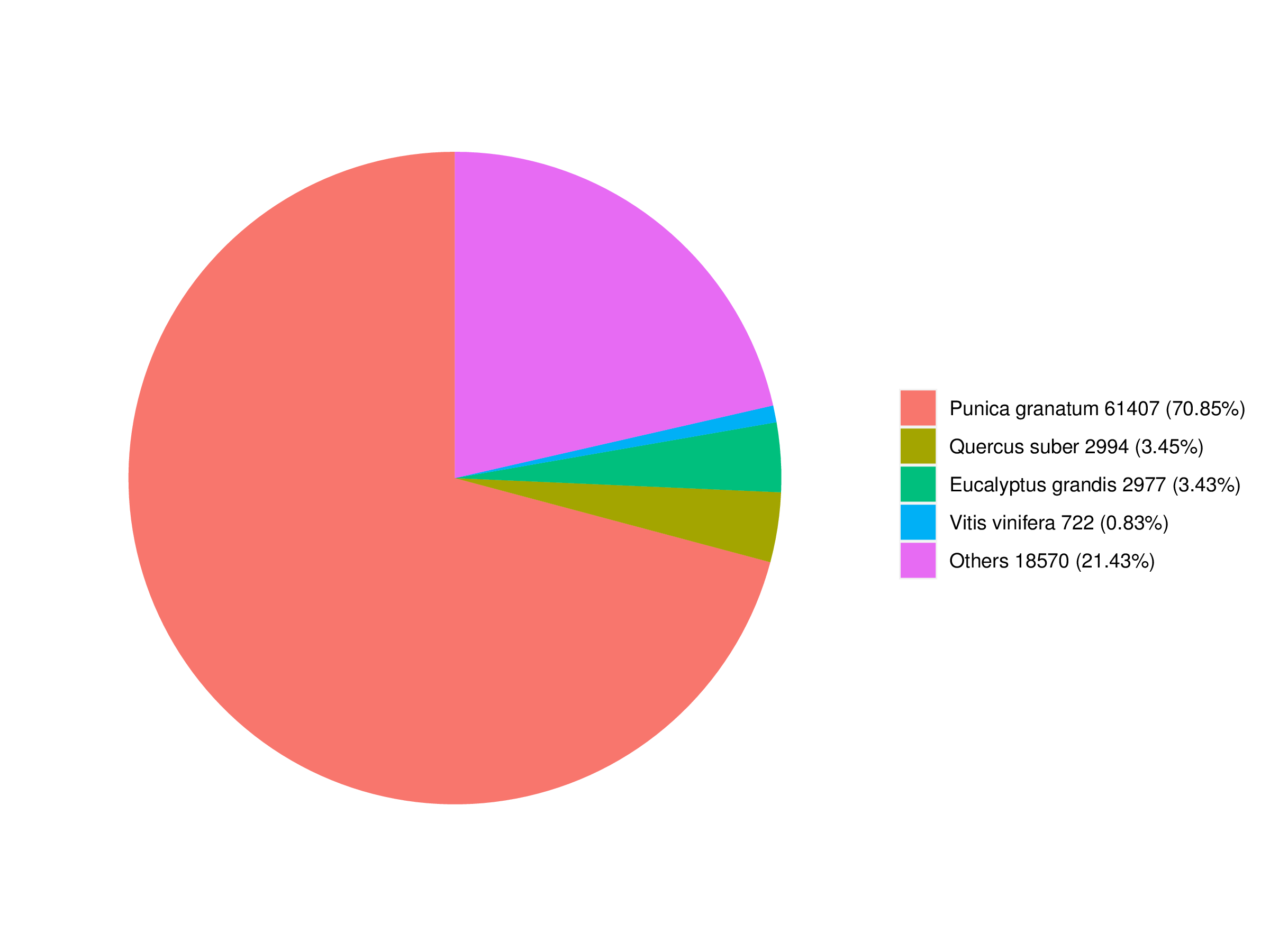


Figure S3 NR database anootation. Similarity of the transcript sequence of *Lagerstroemia indica* to that of allied species.


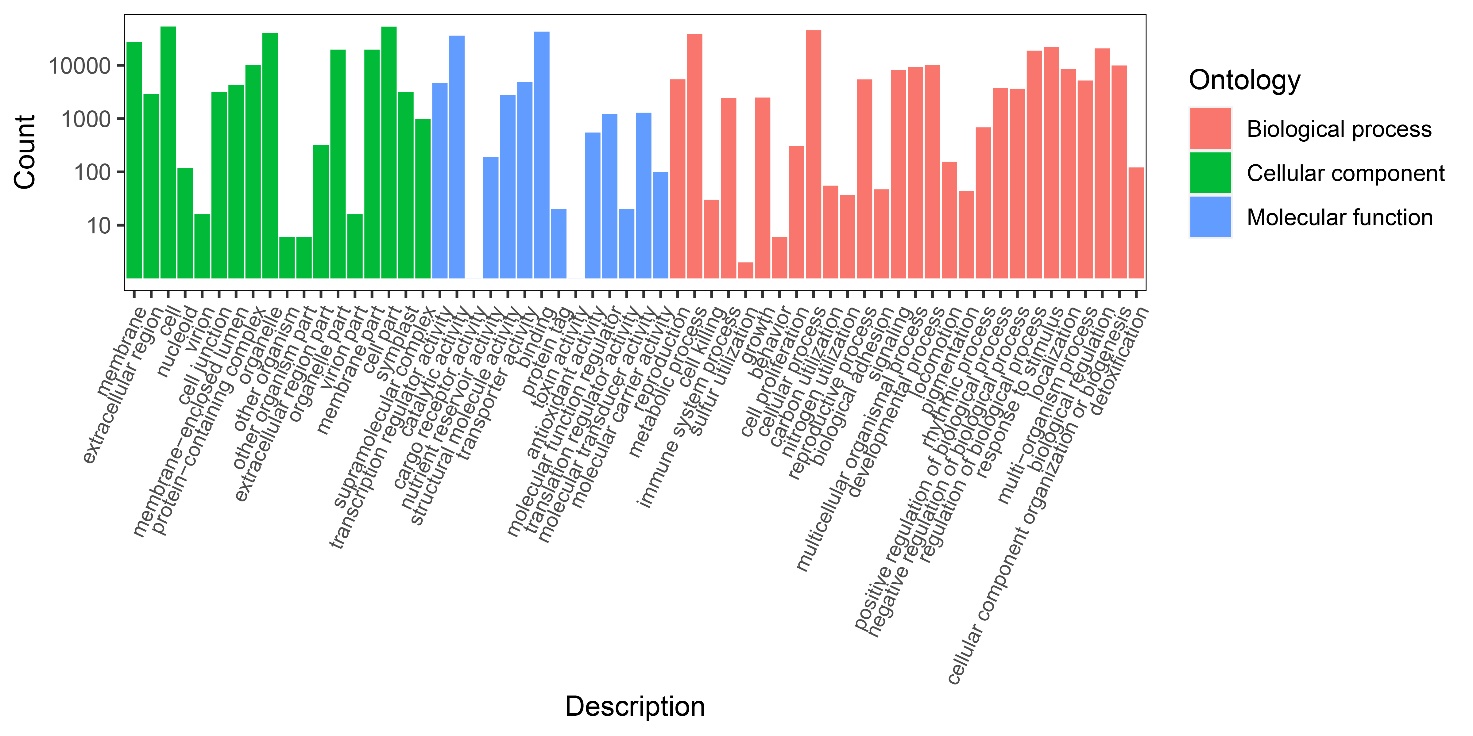


Figure S4 GO database annotation. The founction of unigenes was annotated and categorized into three parts based on GO database.


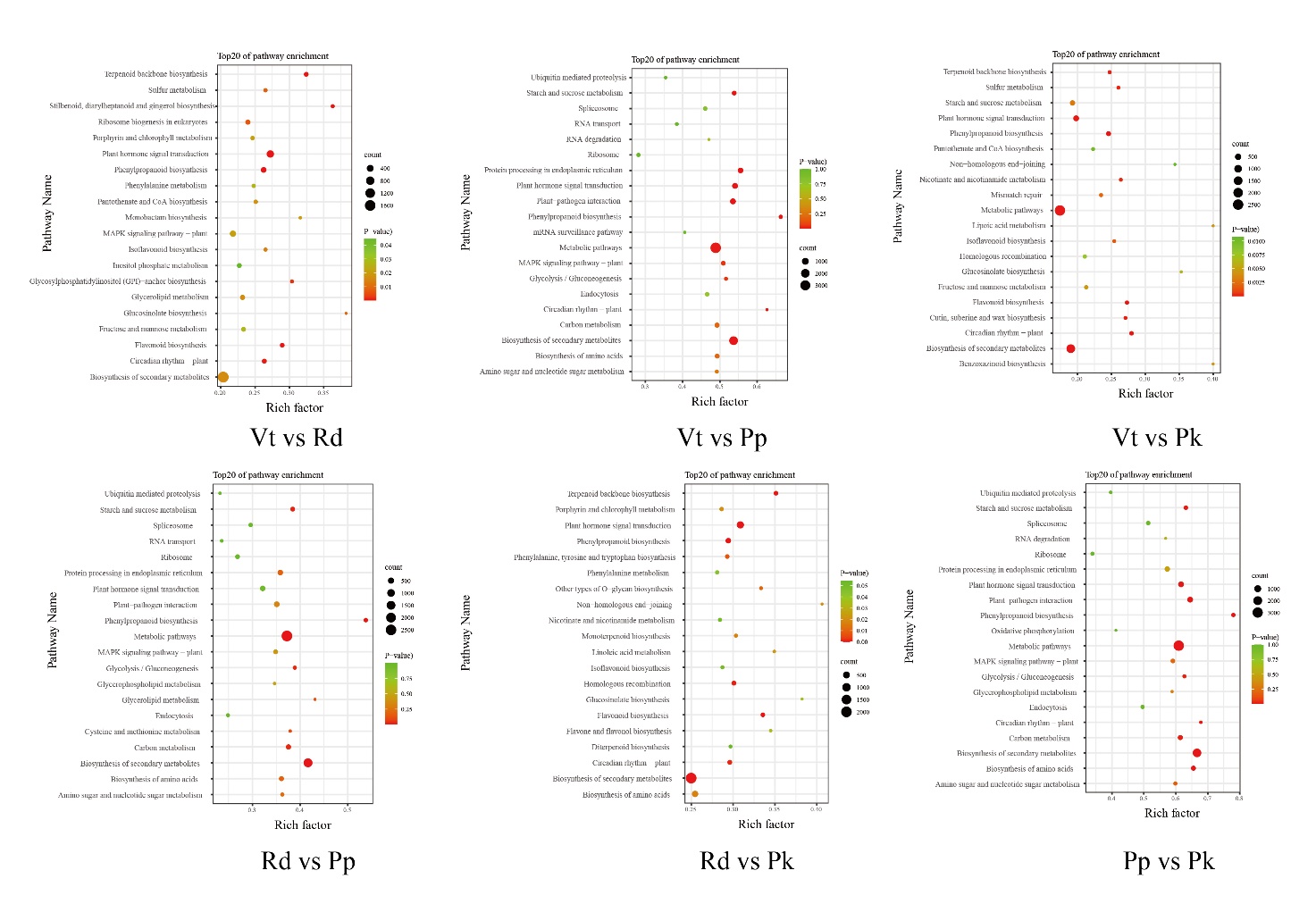


Figure S5 Top 20 KEGG pathway of Vt vs.Rd, Vt vs. Pp, Vt vs.Pk, Rd vs.Pp, Rd vs.Pk, Pp vs.Pk.
