# Supplementary material for: Decoding the formation of diverse petal colors of Lagerstroemia indica by integrating the data from transcriptome and metabolome
Source: Front Plant Sci. 2022 Sep 7;13:970023. doi: 10.3389/fpls.2022.970023 (PMC9490092; doi:10.3389/fpls.2022.970023)
Supplement: Supplementary file 4 [file Table_2.DOCX]

### Method S2

### UPLC Conditions

The sample extracts were analyzed using an UPLC-ESI-MS/MS system (UPLC, SHIMADZU Nexera X2, <https://www.shimadzu.com.cn/>; MS, Applied Biosystems 4500 Q TRAP, <https://www.thermofisher.cn/cn/zh/home/brands/applied-biosystems.html>). The analytical conditions were as follows, UPLC: column, Agilent SB-C18 (1.8 µm, 2.1 mm * 100 mm); The mobile phase consisted of solvent A, pure water with 0.1% formic acid, and solvent B, acetonitrile with 0.1% formic acid. Sample measurements were performed with a gradient program that employed the starting conditions of 95% A, 5% B. Within 9 min, a linear gradient to 5% A, 95% B was programmed, and a composition of 5% A, 95% B was kept for 1 min. Subsequently, a composition of 95% A, 5.0% B was adjusted within 1.1 min and kept for 2.9 min. The flow velocity was set as 0.35 mL per minute; The column oven was set to 40°C; The injection volume was 4 μL. The effluent was alternatively connected to an ESI-triple quadrupole-linear ion trap (QTRAP)-MS.
